# Salivary Melatonin Levels in Comparison with Disease Features and Antihistamine Use: Prospective Follow-Up of Atopic Dermatitis Patients

**DOI:** 10.3390/ijms27209333

**Published:** 2026-10-20

**Authors:** Renata Tomašević, Milena Hanžek, Tamara Sušić, Mirta Peček, Stjepanka Lešić, Liborija Lugović-Mihić

**Affiliations:** 1 Department of Dermatology and Venereology, Sestre Milosrdnice University Hospital Center, Vinogradska Cesta 29, 10000 Zagreb, Croatia; renatavalo@gmail.com; 2 School of Dental Medicine, University of Zagreb, 10000 Zagreb, Croatia; 3 Department of Clinical Chemistry, Sestre Milosrdnice University Hospital Center, 10000 Zagreb, Croatia; milenanjegovan13@gmail.com (M.H.); tami.susic@gmail.com (T.S.); 4 Lipik Special Hospital for Medical Rehabilitation, 34551 Lipik, Croatia; mirta.pec@gmail.com; 5 Department of Dental Medicine, Faculty of Dental Medicine and Health Osijek, Josip Juraj Strossmayer University of Osijek, 31000 Osijek, Croatia; slesic@fdmz.hr

**Keywords:** atopic dermatitis, itching, melatonin, sleep disturbance, antihistamines, treatment, disease severity, Scoring Atopic Dermatitis/SCORAD, Eczema Area and Severity Index/EASI, pruritus

## Abstract

**Background**: Atopic dermatitis (AD) is a common inflammatory dermatosis often accompanied by itching and sleep disturbances. Various treatment options exist, including antihistamines (AHs), even though current clinical recommendations do not mention them. **Methods**: This prospective exploratory study measured and recorded salivary melatonin at two timepoints (T1 and T2) in three groups of participants: those not treated with AHs, those who discontinued H1-AHs, and those for whom H1-AHs were introduced. Also assessed were disease severity (Scoring Atopic Dermatitis/SCORAD and Eczema Area and Severity Index/EASI), pruritus severity (Peak Pruritus Numeric Rating Scale/NRS), and sleep quality (Pittsburgh Sleep Quality Index/PSQI). **Results**: The EASI scores were significantly inversely correlated with salivary melatonin levels (*p* = 0.009) at the first timepoint (T1), but not at the second timepoint (T2). At T2, SCORAD correlated with EASI, NRS, and PSQI (ρ = 0.304–0.842; *p* ≤ 0.022). EASI correlated with NRS and PSQI (ρ = 0.281–0.453; *p* ≤ 0.035). Those who discontinued AHs showed increased AD severity indicators (SCORAD, EASI, NRS), whereas participants for whom AHs were introduced showed decreases in the same scores. After adjusting for baseline values, a significant main effect was detected for the treatment group for T2 SCORAD scores (*p* < 0.001, η^2^ = 0.493). Pairwise comparisons confirmed significant differences among all treatment arms/measures (all *p* values ≤ 0.023). Changes in melatonin levels between the three groups were not significant when baseline SCORAD values were controlled for. **Conclusions**: Our exploratory study observed an inverse association between melatonin levels and AD severity at T1, but this was not replicated at T2. This association should be interpreted with caution due to study limitations. Changes in AD severity and pruritus scores following AH initiation or discontinuation were observed, but the study’s non-randomized design and baseline differences between groups preclude a definitive causal inference, and further research on this topic is needed.

## 1. Introduction

Atopic dermatitis (AD) is a common chronic inflammatory skin disease affecting both children and adults. The main symptom, pruritus, is frequently associated with sleep disturbances and a vicious itch–scratch cycle, where skin inflammation and disease severity worsen as the patient scratches for relief [1,2,3,4]. Persistent pruritus has a profound negative impact on patients’ quality of life, provoking repetitive scratching that compromises skin barrier integrity, promotes cutaneous inflammation and increases the risk of secondary infection. In addition, chronic pruritus is associated with adverse psychosocial outcomes, including impaired concentration, anxiety, and depression [5]. Also, sleep disturbance, as a major clinical AD manifestation, is noted as an important component of disease burden and is incorporated into AD severity assessment using the Scoring Atopic Dermatitis (SCORAD) index [6]. The prevalence of sleep disturbances among AD patients has been estimated at 50–80% in children and 30–90% in adults [7,8]. Sleep disturbances may occur throughout all stages of sleep and include prolonged sleep onset latency, frequent nocturnal waking, difficulty waking up, and excessive daytime sleepiness. However, adequate sleep is essential for maintaining overall health, since both acute and chronic sleep disturbances are associated with a wide range of cognitive, behavioral, and mood impairments. Such disturbances have been linked to poorer academic performance and impose a substantial socioeconomic burden on patients, their families, and society through increased healthcare utilization, higher medical and treatment-related costs, and reduced productivity [9,10].

Melatonin (N-acetyl-5-methoxytryptamine) is a molecule that plays a key role in sleep and sleep disturbances. It is primarily synthesized in the pineal gland from the amino acid tryptophan and is subsequently secreted into the bloodstream, cerebrospinal fluid, and various peripheral tissues, including the retina, bone marrow, gonads, gastrointestinal mucosa, and the skin [11,12,13]. Melatonin secretion follows a circadian rhythm, with nocturnal concentrations approximately tenfold higher than daytime levels. Exposure to light suppresses melatonin secretion, thereby promoting wakefulness, whereas the absence of light during the night increases melatonin production, inducing sleepiness and facilitating sleep onset. Melatonin exerts its biological effects through G protein-coupled membrane receptors expressed in the brain and peripheral organs, as well as through nuclear receptors and calmodulin. Membrane melatonin receptors include two major subtypes: the high-affinity Mel1a receptors (ML1, ML1a, MT1, and MTNR1A), predominantly distributed in the brain and skin, and the low-affinity Mel1b receptors (MT2, ML1b, and MTNR1B), which are distributed throughout various regions of the brain [14,15]. Melatonin regulates sleep by acting on the suprachiasmatic nucleus, the principal circadian pacemaker [16]. Higher serum melatonin levels have been associated with improved sleep quality, reduced sleep fragmentation, and decreased disease severity in conditions such as AD.

Among its various roles, including maintaining skin barrier homeostasis, melatonin is a potent antioxidant. In keratinocytes and mitochondria, melatonin scavenges reactive oxygen and nitrogen species, enhances the activity of antioxidant enzymes, including superoxide dismutase, catalase, and glutathione peroxidase, and increases intracellular glutathione levels [16,17]. During chronic inflammation, characterized by elevated inflammatory mediator levels and reactive free radicals, melatonin suppresses pro-inflammatory pathways by reducing heat shock protein 70 (Hsp70) expression and inhibiting IL-1β, caspase-1, and caspase-3 activity in keratinocytes. It also activates antioxidant enzymes, reduces reactive oxygen species production, inhibits nuclear factor kappa B (NF-κB) translocation, and suppresses 5-lipoxygenase activity, thereby limiting cytokine production and leukocyte migration [16,17]. In addition, melatonin exerts immunomodulatory effects by enhancing both innate and adaptive immune responses through increased cytokine secretion and neutrophil chemotaxis. It upregulates the expression of immune markers, including CD69, CD28, and BCL2, resulting in increased CD4+ T-cell production. Furthermore, increased interleukin-4 (IL-4) promotes a Th2 immune response, and reduced interferon-gamma (IFN-γ) expression suppresses the Th1 response [14,16].

The skin represents a major extrapineal source of melatonin and its metabolites, and studies have demonstrated that melatonin concentrations are significantly higher in the skin than in serum, supporting its autocrine and paracrine activity. Furthermore, the autonomous cutaneous melatonin system comprises various skin cells, including keratinocytes, melanocytes, and fibroblasts, and it contains the enzymes required for melatonin biosynthesis, including tryptophan hydroxylase, arylalkylamine *N*-acetyltransferase, and hydroxyindole-*O*-methyltransferase [18]. For research purposes, melatonin concentrations can be measured in the blood, urine, or saliva [19]. In saliva, melatonin is present exclusively in its free, albumin-unbound form [20]. When measuring melatonin concentrations, both the timing and the sampling method should be carefully considered [14]. Previous studies have shown that salivary and plasma melatonin concentrations may differ by as much as 36%. Salivary melatonin concentrations also exhibit marked circadian variation, typically ranging from 1 to 5 pg/mL during the daytime and from 10 to 50 pg/mL during the night [21,22].

Pruritus in AD is commonly managed with a combination of preventive measures, symptomatic therapies, including emollients and topical or systemic corticosteroids, and immunomodulatory agents. Oral antihistamines (AHs) (H1-AHs) are also frequently prescribed despite limited and conflicting evidence of their clinical efficacy. Although current clinical guidelines for the management of AD do not mention the use of AHs, the literature mentions that short-term use of 1st-generation AHs has been suggested as a useful therapy option for the management of pruritus-related insomnia in AD patients [5,23,24]. A systematic review of 16 studies reported inconclusive evidence supporting the use of AHs in AD patients, although the available data suggest that sedating AHs may improve sleep quality [5,25]. In contrast, the use of 2nd-generation non-sedating AHs is generally not recommended for the management of AD, except in patients with concomitant urticaria or atopy. Although early randomized, double-blind, crossover trials concluded that AHs were ineffective in relieving pruritus associated with AD, the Early Treatment of the Atopic Child (ETAC) trial demonstrated a trend toward less severe pruritus in infants treated with cetirizine compared with placebo; however, this difference did not reach statistical significance [5,26].

Therefore, the aim of the present study was to evaluate melatonin levels in AD patients in relation to the clinical characteristics of the disease and the use of oral H1-AHs (no AH use, introduction of AH therapy, or discontinuation of oral AH therapy) during a prospective follow-up. We hypothesized that AD severity (as measured by SCORAD and the Eczema Area and Severity Index/EASI) is inversely proportional to salivary melatonin levels, and that there is an inverse correlation between disease severity indicators and itching intensity (NRS) and sleep indicators (PSQI). We also wanted to compare results obtained before and after discontinuing AH therapy, i.e., whether discontinuation would cause a significant increase in AD severity (increased SCORAD, EASI) and a decrease in salivary melatonin levels, as well as whether a significant improvement in AD severity (decrease in SCORAD, EASI) and an increase in salivary melatonin would be seen after introducing AH therapy.

## 2. Results

The clinical scores and melatonin levels of study participants, with descriptive statistics at initial measurement (T1), are presented in Table 1.

Most participants were female, with 22/33 (67%) females in the non-AH group, 8/8 (100%) in the “AH discontinued” group and 11/16 (69%) in the “AH introduced” group.

At T1, salivary melatonin was below the lower measurement limit value of 15.63 pg/mL in 17/33 cases (52%) in the non-AH group, 3/8 cases (38%) in the “AH discontinued” group and 9/16 cases (56%) in the “AH introduced” group. Differences between groups were not statistically significant.

### 2.1. Association Between Salivary Melatonin Levels and Clinical Scores for Atopic Dermatitis

#### 2.1.1. Disease Severity (SCORAD, EASI) and Pruritus Intensity (Peak Pruritus Numeric Rating Scale/NRS) in Relation to Salivary Melatonin Levels, Assessed at the First Sampling (T1)

At T1, EASI/disease severity showed a statistically significant inverse monotonic correlation with salivary melatonin levels (ρ = −0.344; *p* = 0.009; Table 2). Melatonin levels were not significantly correlated with either SCORAD or NRS; however, the correlation with NRS approached statistical significance (ρ = −0.248, *p* = 0.063).

#### 2.1.2. Comparison Between Disease Severity Measures (SCORAD, EASI), Pruritus Intensity (NRS), and Sleep Quality (PSQI), Assessed at the Second Sampling (T2)

At T2, SCORAD correlated with EASI (ρ = 0.843; *p* < 0.001), NRS (ρ = 0.304; *p* = 0.022), and the Pittsburgh Sleep Quality Index (PSQI) (ρ = 0.491; *p* < 0.001) (Table 3). EASI correlated with NRS (ρ = 0.281; *p* = 0.035) and PSQI (ρ = 0.453; *p* < 0.001) (Table 3).

### 2.2. Changes in Clinical and Salivary Melatonin Parameters During Prospective Follow-Up in Relation to Antihistamine Use

Our assessment of melatonin levels at T2 showed below-limit values in 14/33 (42%) cases for the non-AH group, 4/8 (50%) cases in the “AH discontinued” group, and 8/16 (50%) cases in the “AH introduced” group. Differences between groups were not statistically significant.

In a secondary, longitudinal analysis, two-way mixed analysis of variance (ANOVA) revealed a significant main effect of time on melatonin levels (*p* < 0.001; η^2^ = 0.408), reflecting an overall temporal variation across the entire cohort; however, the time-by-group interaction was not statistically significant.

In the primary analysis of covariance (ANCOVA) for T2 melatonin levels, after controlling for baseline values of melatonin (T1) as a covariate, there was no significant main effect of group. The primary adjusted analysis showed that the mean difference between the “AH discontinued” group and the non-AH group was −21.83 pg/mL, 95% CI [−148.43, 104.78]. Between the “AH introduced” group and the non-AH group, the mean difference was −12.01 pg/mL, 95% CI [−111.16, 87.13]. For the “AH discontinued” group vs. the “AH introduced” group, the mean difference = 9.81 pg/mL, 95% CI [−131.19, 150.81]. Baseline melatonin levels had a significant influence (*p* < 0.001, η^2^ = 0.271).

A sensitivity analysis with zero-imputation for melatonin values below the lower boundary of the declared measuring range (ANCOVA), and the previously mentioned binary detectability assessment (χ^2^ test), confirmed that in each situation there was no statistically significant main effect of the AH group on T2 melatonin levels, indicating that findings were robust to lower-bound data constraints.

When ANCOVA was conducted to evaluate the changes in melatonin levels across the three groups, controlling for baseline SCORAD values as a covariate, the primary adjusted analysis revealed no statistically significant main effect for the AH group (“AH discontinued” group vs. non-AH group: mean difference = 11.22, 95% CI [−126.05, 148.49]; “AH introduced” group vs. non-AH group: mean difference = 22.31, 95% CI [−96.78, 141.40]; “AH discontinued” group vs. “AH introduced” group: mean difference = 11.09, 95% CI [−147.36, 169.55]).

For clinical AD severity outcomes, primary analyses were based on ANCOVA models adjusted for baseline clinical scores, while two-way mixed ANOVA and unadjusted change scores (ΔT1-T2) were evaluated as secondary longitudinal analyses. Secondary two-way mixed ANOVA for SCORAD showed a statistically significant main effect of time (*p* = 0.026, η^2^ = 0.088) and time-by-group interaction (*p* < 0.001, η^2^ = 0.526). The differences were between the non-AH and “AH introduced” groups (mean difference = −14.10, 95% CI [−24.45, −3.75], *p* = 0.004).

In the primary ANCOVA model for SCORAD at T2, baseline SCORAD values had a significant influence on SCORAD at T2 (*p* < 0.001, η^2^ = 0.947). However, after controlling for baseline values, there was a statistically significant main effect of group on T2 SCORAD levels (*p* < 0.001, η^2^ = 0.493). Significant differences were present between all treatment arms/measures. Specifically, patients in the “AH discontinued” group showed significantly different adjusted mean SCORAD outcomes compared to the non-AH group (mean difference = 7.32, 95% CI [4.18, 10.46], *p* < 0.001) and the “AH introduced” group (mean difference = 10.38, 95% CI [6.75, 14.00], *p* < 0.001). Furthermore, a significant difference was also observed between the “AH introduced” and non-AH groups (mean difference = −3.06, 95% CI [−5.78, −0.33], *p* = 0.023).

Secondary two-way mixed ANOVA results demonstrated that there was no statistically significant main effect of time on EASI (*p* = 0.080, η^2^ = 0.056); however, the time-by-group interaction was statistically significant (*p* < 0.001, η^2^ = 0.447). There was a difference between the non-AH group and the “AH introduced” group (mean difference = −7.99, 95% CI [−13.05, −2.92], *p* = 0.001).

The primary ANCOVA model for EASI at T2 showed that the baseline EASI values had a significant influence on EASI at T2 (*p* < 0.001, η^2^ = 0.938). After controlling for baseline values, there was a statistically significant main effect of group for T2 EASI levels (*p* < 0.001, η^2^ = 0.378). In the primary adjusted analysis, significant differences were observed between the “AH discontinued” group and the non-AH group (mean difference = 3.09, 95% CI [1.46, 4.73], *p* < 0.001) and the “AH introduced” vs. “AH discontinued” groups (mean difference = −4.37, 95% CI [−6.32, −2.42], *p* < 0.001). The adjusted comparison between the “AH introduced” group and the non-AH group was not statistically significant (mean difference = −1.27, 95% CI [−2.73, 0.19], *p* = 0.107).

Secondary two-way mixed ANOVA results showed no statistically significant main effect of time on NRS (*p* = 0.763, η^2^ = 0.002); however, the time-by-group interaction was statistically significant (*p* < 0.001, η^2^ = 0.490). Post-hoc pairwise comparisons revealed that patients in the “AH introduced” group showed significantly different outcomes compared to both the non-AH group (mean difference = 1.28, 95% CI [0.29, 2.27], *p* = 0.007) and the “AH discontinued” group (mean difference = 1.63, 95% CI [0.22, 3.03], *p* = 0.018).

The primary ANCOVA model for NRS at T2 showed that baseline NRS values also had a significant influence on NRS measured at T2 (*p* < 0.001, η^2^ = 0.582). When controlling for baseline values, there was a statistically significant main effect of group on T2 NRS levels (*p* < 0.001, η^2^ = 0.324). Significant differences between individual groups were seen in the primary adjusted analysis: “AH discontinued” vs. the non-AH group (mean difference = 1.82, 95% CI [0.86, 2.78], *p* < 0.001); “AH introduced” vs. the “AH discontinued” group (mean difference = −2.35, 95% CI [−3.57, −1.13], *p* < 0.001). The adjusted comparison between the “AH introduced” group and the non-AH group was not statistically significant (mean difference = −0.53, 95% CI [−1.33, 0.28], *p* = 0.332).

Sleep quality (PSQI), analyzed only at T2, did not differ significantly between the three groups (Table 4).

**Figure 1:**
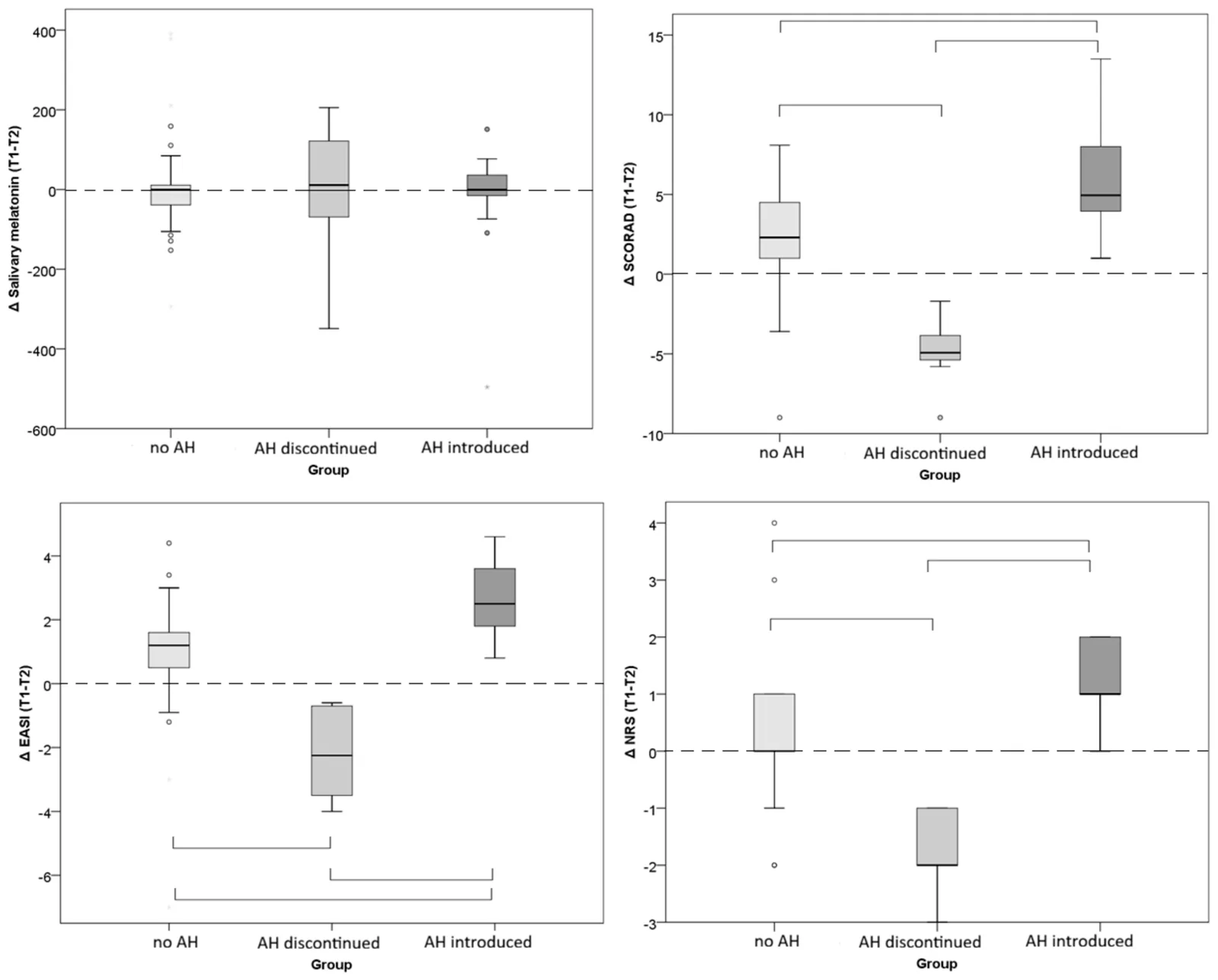
Comparison of changes in salivary melatonin, SCORAD, EASI, and NRS between study groups. The dashed horizontal line indicates the threshold of no change, and horizontal square brackets connect groups which differ significantly.

As part of the secondary longitudinal evaluations, when comparing unadjusted change scores between the timepoints (ΔT1–T2), significant differences between groups were observed for the AD severity indicators, SCORAD, EASI, and NRS (*p* < 0.001; η^2^ = 0.442–0.492), but not for melatonin (Table 5 and Figure 1). Statistically significant differences were found between all three groups. Compared to the non-AH group, the group in which AHs were discontinued showed increased severity (SCORAD, EASI, NRS), and the group in which AHs were introduced showed a reduction in disease severity, i.e., SCORAD, EASI, and NRS scores.

Compared with the non-AH group, discontinuation of AHs was associated with a worsening AD clinical condition, reflected by increased symptom severity, and initiation of AH treatment was associated with an improved clinical condition, reflected in a reduction in symptoms.

## 3. Discussion

Since pruritus impairs the sleep quality of AD patients, melatonin is considered an important molecule in the pathophysiology of AD [27,28]. According to a study by Chang et al., disturbed sleep patterns are common in children with AD, affecting up to 60% of patients, and have a substantial negative impact on quality of life [29]. Among dermatological diseases, melatonin concentrations have been investigated most extensively in AD, but studies have predominantly been conducted in pediatric AD patients. In our exploratory and hypothesis-generation study conducted on adults with AD, AD severity (EASI) significantly inversely correlated with salivary melatonin levels; however, this correlation, which was observed at T1, disappeared at T2. In addition, when comparing the changes between the two measurements/timepoints, significant differences between the groups in which AHs were introduced or discontinued were observed for the AD severity indicators (SCORAD, EASI, and NRS), but not for melatonin. Thus, our patients’ melatonin levels did not significantly correlate with either SCORAD or NRS, although the correlation with NRS approached statistical significance. We also noted that those treated only topically, the non-AH group, also showed a significant decrease in SCORAD and EASI, but not in NRS or melatonin levels. Thus, our study did not provide results which support a clear relationship between AD severity, melatonin, and AH use. To reconcile the statistically significant overall effect of time on melatonin revealed by the mixed ANOVA with the observed clinical and biological stability across treatment groups, several methodological aspects must be considered. While the mixed ANOVA captures aggregate within-subject variance over time, the primary adjusted ANCOVA models (controlling for baseline levels) and secondary longitudinal change-score analyses (ΔT1–T2) revealed no significant differences in melatonin trajectories among the three study arms. The significant overall time effect in the omnibus model therefore reflects broad analytical variance or baseline shifts rather than a directional, treatment-induced modulation of salivary melatonin. However, this absence of a significant (biological) change in melatonin could be interpreted by laboratory aspects of our melatonin analysis—nearly half of our salivary melatonin results were below the lower measurement threshold and were imputed as one identical value. This extensive left-censoring may materially affect rank correlations and paired analyses and therefore limits confidence in conclusions regarding the longitudinal biological stability of salivary melatonin. Analytical sensitivity, limit of detection (LOD), lower limit of quantification (LLOQ), and measuring range are distinct analytical characteristics; however, for the commercially available Human Melatonin ELISA Kit used in this study (catalogue no. A327018, Antibodies.com Limited, Cambridge, United Kingdom), the manufacturer provides only analytical sensitivity and the measuring range. Specifically, the declared measuring range is 15.63–1000 pg/mL, while the analytical sensitivity is 9.38 pg/mL. Separate values for the LOD and LLOQ are not reported in the manufacturer’s documentation and therefore could not be provided or independently inferred. So, this assay may not have been sufficiently sensitive for this study population. Other potential reasons for the dissociation between clinical AD improvement/worsening and stable salivary melatonin could be melatonin-independent symptom changes, inadequate sensitivity of a single salivary sample, circadian and pre-analytical variability, limited statistical power, and extensive censoring of melatonin results. In addition, we analyzed PSQI only at T2; thus, any changes in patients’ sleep quality over the course of the treatment period could not be demonstrated.

The first study evaluating melatonin levels in AD was conducted by Schwarz et al. in 1988, who assessed serum melatonin levels in adult patients with severe AD [30]. Measurements were obtained every two hours over a 24-h period, which showed predominantly reduced melatonin levels and an altered nocturnal melatonin secretion pattern, suggesting there is a disruption of circadian melatonin regulation in severe AD [30]. In other studies, an association between lower melatonin levels and greater AD disease has been consistently demonstrated. Jaworek et al. reported significantly lower serum melatonin concentrations in patients with very severe AD compared to those with less severe AD [11]. Similarly, Dwiyana et al. found significantly reduced serum melatonin levels in children with moderate AD compared with healthy controls [31]. In addition to serum measurements, melatonin has also been assessed in saliva. Kimata demonstrated significantly lower nocturnal salivary melatonin concentrations in 40 children with mild to moderate AD compared to healthy controls (26.5 pg/mL vs. 58.6 pg/mL) [32].

In the research literature, melatonin has been increasingly recognized for its potential role in mitigating AD and other allergic inflammatory diseases through multiple biological mechanisms. Cutaneous melatonin synthesis is regulated by the circadian rhythm and is upregulated in response to oxidative and environmental stress, thereby supporting the skin’s adaptation to fluctuations in the light–dark cycle [18]. The potential role of melatonin in AD is further supported by its well-established anti-inflammatory and immunomodulatory properties. Melatonin contributes to skin barrier integrity preservation by limiting the chronic activation of inflammatory pathways. In particular, it inhibits activation of the transcription factor NF-κB, thereby reducing the expression of pro-inflammatory cytokines, including IL-1β and IL-6, as well as inducible nitric oxide synthase (iNOS) and cyclooxygenase-2 (COX-2) [18]. Furthermore, melatonin attenuates caspase activation, thereby limiting stress-induced apoptosis and inflammation and protecting against skin barrier dysfunction associated with chronic inflammation and oxidative stress. Melatonin also exerts broad immunomodulatory effects by influencing both innate and adaptive immune responses. It regulates T-cell differentiation and activation, promotes the production of IFN-γ and IL-2, and contributes to Th1, Th2, and Th17 immune response balance. In addition, melatonin enhances the activity of regulatory T cells (Tregs), thereby promoting immune tolerance and preventing excessive inflammatory responses [15,18,33]. Melatonin modulates the secretion of both pro-inflammatory and anti-inflammatory cytokines and influences the proliferation and differentiation of immune cells [14]. Experimental studies further suggest that melatonin may ameliorate AD-associated inflammation by reducing total serum IgE and IL-4 levels [34]. Considering the close functional relationship between the cutaneous immune system, which is important in AD, and the epidermal barrier, melatonin immunomodulatory effects may further contribute to the maintenance of barrier integrity by limiting uncontrolled inflammatory activation and preventing tissue damage [18]. As is known, AD patients have a compromised skin barrier and often experience delayed skin healing, a prolonged inflammatory phase, and repetitive scratching due to itching. Consequently, due to genetic and structural defects, the skin fails to properly regenerate its outer layer. High levels of Th2-mediated immune signals, such as IL-4, impair keratinocyte recovery and slow down tissue repair. In the itch-scratch cycle, damaged skin easily accumulates microorganisms (e.g., *Staphylococcus aureus*), leading to secondary infections, while scratching disrupts tissue healing, deepens wounds, and triggers further inflammation [35,36,37,38]. Therefore, according to the literature, when looking for an effective therapy for AD patients, different aspects of the disease and AD-related conditions should be taken into account, and to develop more therapy options and a multidisciplinary approach, additional research is needed [38].

In addition, the skin exhibits circadian fluctuations in proliferative, reparative, and immune functions that are regulated by peripheral biological clocks, including clock genes such as *PER1*. Melatonin synchronizes these local circadian oscillators, thereby supporting epidermal homeostasis and tissue repair [18]. Emerging evidence also suggests that melatonin may influence the skin microbiome by modulating microbial composition, metabolite production, and epithelial barrier integrity, giving rise to the concept of the melatonin–circadian rhythm–skin microbiome axis. Collectively, melatonin’s antioxidant, anti-inflammatory, immunomodulatory, and anti-apoptotic properties, together with its potential effects on skin tight junction proteins and circadian regulation, support its role as a promising mediator of skin barrier homeostasis at both the cellular and molecular levels [1,11,18].

While previous clinical studies on melatonin and AD primarily focused on children, multiple significant human studies have specifically analyzed melatonin oral supplementation in adult patients. According to a recent systematic review with meta-analysis comparing melatonin supplementation (3–6 mg/day) with placebos, melatonin significantly reduced sleep-onset latency and improved clinical disease severity, while maintaining a favorable safety profile [1]. (Melatonin primarily facilitates sleep initiation rather than increasing total sleep duration) [1,39,40]. A significant improvement in SCORAD has also been reported when comparing supplementation with placebos—following 6 weeks of melatonin supplementation in one study, SCORAD scores and serum total IgE levels improved, though without significant impact on pruritus scores, high-sensitivity C-reactive protein, sleep-onset latency, or total sleep time [41]. According to studies on children with AD, oral melatonin reduced sleep-onset latency by 21.4 min, compared to placebos, and decreased AD severity, as assessed by SCORAD, without adverse effects [11,31,42,43]. As available literature has reported that melatonin may improve AD condition, melatonin may represent a promising therapeutic option for preserving skin integrity and maintaining epidermal barrier function [14]. According to previous study results, systemic melatonin may be considered as an adjunctive therapy for select patients, but routine use cannot be recommended currently due to limited clinical evidence. Thus, larger, well-designed randomized controlled trials are needed to establish melatonin’s efficacy and safety [1,11,31,44].

Still, H1-AHs continue to be widely prescribed for AD despite conflicting evidence of their clinical efficacy [4,45,46,47]. Despite systematic reviews which indicate that a histamine receptor blockade does not substantially improve AD-related pruritus or inflammation, and insufficient current evidence to recommend routine H1-AH therapy for AD, several studies have reported its potential clinical benefits. For example, cetirizine has been shown to reduce the need for topical corticosteroids, while combined H1R and H4R antagonism produced synergistic anti-inflammatory and antipruritic effects comparable to those of prednisolone [5,45,46]. Moreover, treatment with the AH fexofenadine resulted in a modest but significant reduction in AD-associated pruritus compared with placebo, with improvement observed as early as the first day of treatment, suggesting that histamine acting through H1 receptors contributes, at least in part, to pruritus occurrence/induction in AD. Our exploratory study recorded that an introduction of AHs was related to an improvement in AD manifestations/symptoms, while discontinuation often led to deterioration. We must mention that our patients who discontinued AHs initially had more severe AD, which may have influenced the final results. Concerning the literature data, one study demonstrated that AHs may influence specific components of SCORAD, including significant patient-reported clinical improvements in pruritus and sleep disruptions, but without significant improvements in dermatologist-recorded SCORAD components (eczema extent and intensity). Still, this shows a favorable trend, which may reflect that histamine plays a (limited) role in these disease domains [5]. Several studies have supported the efficacy of AHs, particularly second-generation agents, in reducing pruritus and sleep disturbances in AD. Compared with first-generation AHs, second-generation agents have improved therapeutic indices and safety profiles, with minimal effects on cognitive function and sedation due to their limited penetration of the blood–brain barrier. Furthermore, their longer action duration allows less frequent administration. Notably, cumulative use of first-generation AHs has been associated with an increased risk of cognitive impairment and dementia. [5,48]. Most second-generation AHs exhibit higher selectivity for H1 receptors, resulting in fewer anticholinergic adverse effects (tachycardia, xerosis, delirium, etc.), which further supports their suitability for AD management [5]. Some literature data has mentioned that AHs appear to be beneficial primarily through the reduction in pruritus and sleep disturbances, interrupting the itch–scratch cycle. In addition, their use may reduce the need for other topical therapies, such as corticosteroids, which are effective in controlling AD but may be associated with adverse effects (skin atrophy, exacerbation of pre-existing infections, contact dermatitis, etc.).

One of the main strengths of this study is its prospective design and that it is the first evaluation of salivary melatonin levels in adult AD patients. Coupled with this, our study is also the first to simultaneously compare patients not using AH therapy with those in whom AH treatment was discontinued or introduced during follow-up. Salivary melatonin assessment offers several practical advantages over serum measurements: saliva collection is non-invasive, safer, and easier to perform repeatedly, and provides similar accuracy for the onset of melatonin action in low light [49]. Although our findings provide additional insight into the potential interplay between AH therapy, disease-related symptoms, and melatonin regulation in AD, a few study limitations should be considered. This non-randomized study involved groups that differed clinically at baseline; e.g., patients who started AHs had significantly higher AD severity scores than the other groups. Furthermore, observed changes in condition—i.e., worsening after AH discontinuation and improvement after introducing AH—may represent an observational finding rather than evidence of AH treatment efficacy. Moreover, only a single saliva sample was collected at each measurement point, whereas collecting multiple samples (during the evening hours), as well as accounting for more detailed seasonal and other factors (light exposure, screen use, sleep schedule, etc.), would have provided better insight into melatonin levels. Additionally, a high proportion of melatonin values fell below the lower boundary of the declared measuring range, which limits confidence in rank-based correlations, paired analyses, and conclusions regarding longitudinal biological stability. We can also note the limited generalizability of the data, as this was a single center study with predominantly female participants, a 2-week timeframe, and no objective real-time sleep assessment (PSQI evaluates sleep quality/habits over the preceding month). It is also possible that patients did not follow instructions exactly or did not admit to not following certain instructions. Finally, the relatively small sample sizes of the individual study groups (especially the “AH discontinued” group) may have limited the statistical power and the generalizability of the findings. Larger, well-designed prospective studies would be needed to compare observations. Overall, our findings may be a basis for further studies. Although current guidelines do not support H1-AH monotherapy for AD-associated pruritus, according to some literature data, AHs may provide additional benefits through reduction in the itch–scratch cycle intensity and modulation of neural sensitization. For instance, according to literature data, the anti-inflammatory effects of topical corticosteroids may act synergistically with antihistaminic mechanisms, contributing to improved symptom control [50]. Further clinical studies are needed to better define the subgroup of AD patients who may benefit most from oral AH therapy and to clarify its long-term therapeutic potential.

## 4. Materials and Methods

### 4.1. Participant Recruitment and Eligibility

This prospective study was conducted at the Department of Dermatovenereology, University Hospital Center “Sestre Milosrdnice”, Zagreb, Croatia, between February 2025 and April 2026 and was approved by the Ethics Committee of the University Hospital Center “Sestre Milosrdnice”, Zagreb, Croatia, in January 2025 (protocol number: 251-29-11/3-24-11). All patients included in the study gave their written informed consent to participate.

AD patients were enrolled in the study according to the following inclusion criteria: a diagnosis of AD based on the Hanifin and Rajka diagnostic criteria and age ≥ 18 years [47,48,49]. We excluded AD patients who had received systemic corticosteroids and/or immunosuppressive, psychoactive, or biological therapy within the two weeks preceding study entry, as well as those who used drugs that could potentially influence itch and sleep. We also excluded patients with a recent or current exacerbation of allergic asthma, rhinoconjunctivitis, or seasonal allergic rhinitis, as well as pregnant or breastfeeding women, smokers, and individuals working in shifts because of the potential impact on sleep quality. Additional exclusion criteria included the presence of oral diseases (oral candidiasis, other oral infections, oral lichen planus, oral injuries, or periodontal disease), severe chronic diseases (type 1 or type 2 diabetes mellitus, arterial hypertension, autoimmune diseases, or malignancies), the use of dentures or orthodontic appliances, and the inability to understand the nature and purpose of the study or the informed consent document. To exclude oral conditions and diseases, all participants underwent an oral examination performed by a dentist and/or a specialist for oral diseases. Finally, if a participant took prohibited medications for any reason during the study, he/she was then subsequently excluded from the study. Thus, only those who were assessed at both T1 and T2 were included in the final analysis.

Eligible study participants were identified among patients who had come to our hospital clinic for an initial dermatological examination. We invited them to participate in the study after asking questions about their therapy use during the previous few weeks; i.e., their use of AHs determined their potential involvement and group assignment. Due to established primary care practices for AD treatment, many AD patients take AHs unrelated to allergies; thus, at the time our patients arrived for a dermatological examination at our clinic (tertiary care center), many of them were already taking AHs. For greater study group homogeneity, only those taking desloratadine or bilastine, one tablet daily, were invited to participate in the study. We excluded those AD patients who were taking AHs to treat a current allergic condition. Alongside those who took AHs, there were also AD patients who did not. In line with our study’s objective—to examine the impact of AH use on the status of AD—we offered the introduction of AH therapy to AD patients who were not already taking them, and some agreed to start taking them for the study (Group III). Others continued not to take AHs and also agreed to be involved in the study (Group I).

### 4.2. Study Design and Participants

This prospective study, which included two timepoint assessments (T1 and T2) of the analyzed variables, comprised three groups of adult patients with AD: (I) patients not receiving AH therapy, (II) patients in whom AH therapy (10 mg of bilastine or 5 mg of desloratadine) was discontinued, and (III) patients in whom AH therapy was introduced (10 mg of bilastine or 5 mg of desloratadine). We checked their potential current allergy and excluded those AD patients who were taking AHs to treat a current allergic condition (respiratory, etc.).

Those who agreed to participate in the study received instructions regarding further AH use according to study protocol. Each patient was provided their own specific dates for their T1 and T2 assessments. Patients also received a sheet with a daily therapy chart that they were required to complete throughout the study’s duration. The study’s main author regularly reminded them of this requirement and those who failed to comply were excluded. The lead author also ensured patient adherence by providing participants with instructions and continuously checking patient AH use, as well as the use of any other systemic or topical therapies. (The regularity of daily AH use was monitored during the two weeks prior to timepoint T1).

After collecting a satisfactory number of patients, enough for statistical analysis, we stopped recruiting. In the end, the study included 57 adult patients with AD (41 women and 16 men), aged 20–53 years. All patients maintained constant contact with the lead study author throughout the study period, who checked their therapy use.

Patients in **Group I** remained without AH therapy throughout the study, including the two weeks preceding enrollment and the two-week interval between the first and second study visits, which is in accordance with current AD treatment guidelines, which do not recommend routine AH therapy.

Patients in **Group II** had regularly been receiving AH therapy the two weeks prior to study enrollment (10 mg of bilastine or 5 mg of desloratadine), one tablet every day, documented on paper; however, they were instructed to discontinue treatment immediately after the first study assessment (T1) and to remain without AH therapy until the second study visit (T2) two weeks later. Given that the half-life of AHs is short (up to 2 days—specifically 12 to 14.5 h for bilastine and 27 h for desloratadine) and that they are eliminated from the body within 3 days, any AHs taken prior to the study had no impact on the measured parameters, considering the two-week period of controlled abstinence.

Patients in **Group III** initiated second-generation AH therapy (10 mg of bilastine or 5 mg of desloratadine) immediately after the first study assessment and continued treatment for the subsequent two weeks until the second study visit. The regularity of their daily AH use was also monitored during this time by the main author, who was in continual contact with them and checked their use according to the study’s adherence requirements.

Before the first measurement (T1), participants received detailed information about the study, including instructions regarding the prescribed topical treatment regimen, including the frequency and method of application, comprehensive instructions on questionnaire completion, saliva collection, and sample storage. During the study, participants were allowed to use topical corticosteroid therapy (betamethasone and alclometasone dipropionate) and regular skin care emollients, provided that the treatment regimen remained stable throughout the entire study period from the initial visit, the first (T1) study visit, to the second study visit (T2), without the introduction of any new medications that could affect AD. According to the study protocol, a topical corticosteroid was used proactively (twice weekly on recurring problem areas, with 12-h intervals (morning and evening applications). Specifically, for lesions on the trunk and limbs betamethasone (betametazondipropionat) was used, while for lesions on certain sensitive regions (face and flexures) alclometasone dipropionate was recommended. Emollients were regularly used twice daily in the periods between steroid applications. During the study, some patients required systemic immunosuppressant therapy due to an insufficient response to topical therapy, so they were then excluded from the study.

At the first study visit (T1), all participants underwent the first assessment of salivary melatonin levels and clinical parameters related to AD severity and sleep. Most patients belonged to Group I and remained without AH therapy throughout the study (N = 33). In Group II, second-generation AH therapy (10 mg of bilastine or 5 mg of desloratadine) was discontinued after the first study visit (N = 8). In Group III, second-generation AH therapy (bilastine or desloratadine, one tablet daily) was initiated immediately after the first study assessment and continued until the second study visit (N = 16).

Two weeks after the baseline assessment, all participants underwent a second evaluation of the same study parameters (T2). At both study visits, salivary melatonin samples were collected, and disease severity was assessed using the SCORing Atopic Dermatitis (SCORAD) index and the Eczema Area and Severity Index (EASI). Pruritus intensity was evaluated using the Peak Pruritus Numerical Rating Scale (Peak Pruritus NRS) [51,52,53]. The participants were examined and assessed consistently by the same person on our dermatology team. Sleep quality was assessed only at the second study visit using the Pittsburgh Sleep Quality Index (PSQI), as this validated questionnaire evaluates sleep quality and sleep habits over the preceding month, corresponding to the overall study period from baseline to the second assessment [54,55].

### 4.3. Salivary Melatonin Analysis

Participants were instructed to refrain from eating and drinking for at least two hours before saliva collection. Foods with a high sugar content, acidic foods, and caffeine were avoided, as these can lower salivary pH. They were advised to avoid cigarette smoking and alcohol consumption for at least 12 h before sampling. In cases of mucosal damage and/or oral cavity infection, saliva sampling was postponed. Saliva sampling was performed in a standing position at 11:00 PM during spring and summer and at 10:00 PM during autumn and winter.

Saliva collection kits were provided to participants during their initial visit, together with written instructions covering restrictions regarding food, beverages, alcohol consumption, smoking, and appropriate oral hygiene before saliva collection.

Following collection, saliva samples were kept on ice until analysis to minimize bacterial and protease activity. Samples were stored in a refrigerator at 2–8 °C for a maximum of 72 h before being transferred to storage at −80 °C, where they remained until analysis. On the day of analysis, the samples were thawed and vortex-mixed at room temperature and then centrifuged at 1500× *g* for 10 min.

Melatonin concentrations were determined using a competitive enzyme-linked immunosorbent assay (ELISA) (Human Melatonin ELISA Kit, catalogue no. A327018, Antibodies.com Limited, Cambridge, United Kingdom) according to the manufacturer’s instructions. The declared measuring range of melatonin was 15.63–1000 pg/mL and the analytical sensitivity was 9.38 pg/mL. The intra-assay coefficient of variation (CV) was <7%, and the inter-assay CV was <9%.

### 4.4. Clinical Outcome Measures (Scores)

Disease severity was assessed using validated clinical scoring systems, including the SCORing Atopic Dermatitis (SCORAD) index and the Eczema Area and Severity Index (EASI) [51,52,53].

The SCORAD index assessed the extent of disease, intensity of clinical signs, and subjective symptoms, including pruritus and sleep disturbance during the previous three days. Subjective symptoms were evaluated using a visual analogue scale ranging from 0 to 10 (0 indicating no pruritus and 10 indicating maximum pruritus intensity). The total SCORAD score was calculated by combining the scores for disease extent, intensity of clinical signs, and subjective symptoms, with a maximum possible score of 103. Disease severity according to SCORAD was classified as mild AD (<25 points), moderate AD (25–50 points), and severe AD (>50 points) [52,53].

The EASI was used to evaluate disease extent across four anatomical regions (head and neck, trunk, upper extremities, and lower extremities) and to assess four clinical signs: erythema, induration/papulation, excoriation, and lichenification, each graded on a scale from 0 to 3. Disease severity according to the EASI score was categorized as clear (0), mild (0.1–5.9), moderate (6.0–22.9), and severe (23.0–72) [51]. Unlike SCORAD, EASI does not include subjective symptoms such as pruritus and sleep disturbance.

At both study assessments, patients were asked to rate the intensity of pruritus experienced during the previous 24 h using an 11-point numerical rating scale ranging from 0 (no itch) to 10 (worst itch imaginable). This measure represented the Peak Pruritus Numerical Rating Scale (Peak Pruritus NRS) [54].

Sleep quality was assessed only at T2 using the PSQI, as this instrument evaluates sleep quality and sleep habits during the preceding month. The PSQI consists of seven components assessing subjective sleep quality, sleep latency, sleep duration, habitual sleep efficiency, sleep disturbances, use of sleep medication, and daytime dysfunction. A global PSQI score of >5 indicates clinically significant sleep disturbance [55].

### 4.5. Statistical Analysis

Salivary melatonin concentrations below the assay detection limit (15.63 pg/mL) were imputed as half the lower boundary of the declared measuring range (7.82 pg/mL) for statistical analyses. This is a standard, widely accepted conservative practice in biomedical research aiming to prevent data loss and selection bias while maintaining statistical power without arbitrarily assigning zero or the full threshold value.

The normality of data distribution was assessed using the Kolmogorov–Smirnov test. Correlations between salivary melatonin levels, disease severity, and sleep quality were evaluated using Spearman’s rank correlation coefficient. Differences in melatonin levels and disease severity between the two timepoints were compared using the Wilcoxon signed-rank test. The effect size for the Wilcoxon test was calculated using the formula r = Z/√N and interpreted according to Cohen’s criteria as small (0.10–0.29), medium (0.30–0.49), or large (>0.50). Differences among the three groups were analyzed using the Kruskal–Wallis test, followed by the Mann–Whitney test with Bonferroni correction as a post-hoc test. Effect size was quantified as η^2^ using the formula η^2^ = (H − k + 1)/(n − k) and interpreted according to the squared values of Cohen’s r criteria. Frequencies were compared by the χ^2^ test, and Cramer’s V was used for effect size.

Longitudinal changes and interaction effects between time and treatment groups were evaluated using a two-way mixed analysis of variance (ANOVA), with time as the within-subjects factor, and the AH group as the between-subjects factor. Effect sizes were reported as partial η^2^. Furthermore, an analysis of covariance (ANCOVA) was conducted to evaluate group differences at T2, controlling for baseline values as covariates. For this, the Bonferroni post-hoc test was used.

The 95% confidence intervals (95% CI) for the median changes in melatonin levels and disease severity were estimated using bootstrapping with 1000 resamples. Statistical analyses were performed using IBM SPSS Statistics for Windows, Version 22.0 (IBM Corp., Armonk, NY, USA).

## 5. Conclusions

Our exploratory study observed an inverse association between melatonin levels and AD severity at the first timepoint, but this was not replicated at the second timepoint. This result should be interpreted with caution due to study limitations. Also, we found that introducing or discontinuing H1-AH led to changes in AD severity/pruritus indicators, but the non-randomized study design and baseline differences between groups preclude a definitive causal inference. Although current guidelines do not mention/support H1-AH monotherapy for AD-associated pruritus/inflammation, our results indicate that this relationship/treatment possibility needs to be further explored. Larger studies, with greater sample sizes and with more details related to AD condition follow-up, should explore whether AHs could provide additional benefits in the reduction in clinical AD features together with the anti-inflammatory effects of topical corticosteroids.

## Figures and Tables

**Table 1:** Descriptive statistics for clinical scores and salivary melatonin at the first sampling (T1).

Variable	Group	Mean ± Std. Dev.	Median (Interquartile Range)	Min–Max
Age (years)	No AH (N = 33)	31.1 ± 8.2	20–49	30 (24–37)
	AH Discontinued (N = 8)	30.2 ± 11.8	20–53	26 (22–40)
	AH Introduced (N = 16)	32.4 ± 10.7	20–53	30 (24–39)
Salivary melatonin (pg/mL)	No AH (N = 33)	89.81 ± 159.46	7.82 (7.82–99.10)	7.82–722.89
	AH Discontinued (N = 8)	112.90 ± 124.81	78.27 (7.82–192.95)	7.82–353.81
	AH Introduced (N = 16)	38.15 ± 50.47	7.82 (7.82–56.11)	7.82–194.85
SCORAD	No AH (N = 33)	30.26 ± 8.55	28.85 (23.45–35.15)	15.40–58.85
	AH Discontinued (N = 8)	31.8 ± 14.2	25.8 (20.8–47.4)	16.5–53.6
	AH Introduced (N = 16)	46.1 ± 21.0	36.3 (29.9–67.7)	26.8–93.3
EASI	No AH (N = 33)	12.14 ± 5.0	11.4 (9.3–14.5)	5.8–30.6
	AH Discontinued (N = 8)	11.8 ± 8.0	7.8 (5.2–20.5)	4.6–23.3
	AH Introduced (N = 16)	21.0 ± 9.4	18.5 (14.3–29.3)	10.2–41.6
NRS	No AH (N = 33)	4.2 ± 1.2	4 (3–5)	2–7
	AH Discontinued (N = 8)	2.75 ± 0.46	3.00 (2.25–3.00)	2–3
	AH Introduced (N = 16)	5.88 ± 1.93	5.50 (4.25–8.00)	3–9

**Table 2:** Association between melatonin levels and disease severity (SCORAD, EASI and NRS) for the total sample (N = 57) at T1, assessed by Spearman’s rank correlation.

Variable		Melatonin (pg/mL) T1	SCORAD T1	EASI T1	NRS T1
Melatonin (pg/mL) T1	ρ	1	−0.229	−0.344	−0.248
	*p*		0.087	0.009	0.063
SCORAD T1	ρ	−0.229	1	0.880	0.439
	*p*	0.087		<0.001	0.001
EASI T1	ρ	−0.344	0.880	1	0.463
	*p*	0.009	<0.001		<0.001
NRS T1	ρ	−0.248	0.439	0.463	1
	*p*	0.063	0.001	<0.001	

**Table 3:** Association between melatonin, disease severity, and sleep quality for the total sample (N = 57), assessed at the second sampling (T2) by Spearman’s rank correlation.

Variable		Melatonin (pg/mL) T2	SCORAD T2	EASI T2	NRS T2	PSQI T2
Melatonin (pg/mL) T2	ρ	1	−0.021	−0.086	0.036	0.139
	*p*		0.874	0.523	0.791	0.302
SCORAD T2	ρ	−0.021	1	0.843	0.304	0.491
	*p*	0.874		<0.001	0.022	<0.001
EASI T2	ρ	−0.086	0.843	1	0.281	0.453
	*p*	0.523	<0.001		0.035	<0.001
NRS T2	ρ	0.036	0.304	0.281	1	0.363
	*p*	0.791	0.022	0.035		0.005
PSQI T2	ρ	0.139	0.491	0.453	0.363	1
	*p*	0.302	<0.001	<0.001	0.005	

**Table 4:** Comparison of sleep quality between groups at T2.

	Group			*p* *	Effect Size
Variable	Non-AH (N = 33)	AH Discontinued (N = 8)	AH Introduced (N = 16)		
PSQI T2 (median and IQR)	4 (3–4)	4 (3.3–6.3)	6 (3.3–13.8)	0.111	0.045
PSQI T2 > 5	9/33 (27.3%)	2/8 (25%)	8/16 (50%)	0.247	0.221

* For PSQI values, the Kruskal–Wallis test was used with η^2^ for effect size. For PSQI categories χ^2^ test was used with Cramer’s V for effect size.

**Table 5:** Comparison of changes in disease severity and melatonin (ΔT1–T2) between groups (median and 95% CI). Unadjusted change scores are presented.

Variable	Non-AH (N = 33)	AH Discontinued (N = 8)	AH Introduced (N = 16)	*p*	r
Δ Melatonin T1–T2	0 (−8.63–0)	11.17 (−137.75–160.32)	0 (−15.22–35.94)	0.780	0.009
Δ SCORAD T1–T2	2.3 (1.5–4) ^a^	−4.9 (−5.8–(−3.5)) ^b^	5 (4–8) ^c^	<0.001	0.442
Δ EASI T1–T2	1.2 (0.6–1.6) ^a^	−2.3 (−4–(−0.6)) ^b^	2.5 (1.8–3.6) ^c^	<0.001	0.471
Δ NRS T1–T2	0 (0–1) ^a^	−2 (−2–(−1)) ^b^	1 (1–2) ^c^	<0.001	0.492

*p*-level of significance for the Kruskal–Wallis test, r-effect size. For each row, groups that do not share the same superscript letters differ significantly according to the Mann–Whitney test with Bonferroni correction. The 95% CI for the median changes in melatonin levels and disease severity were estimated using bootstrapping with 1000 resamples.

## Data Availability

The original contributions presented in this study are included in the article. Further inquiries can be directed to the corresponding author.
